# Designing MPAs for food security in open-access fisheries

**DOI:** 10.1038/s41598-019-44406-w

**Published:** 2019-05-29

**Authors:** Reniel B. Cabral, Benjamin S. Halpern, Sarah E. Lester, Crow White, Steven D. Gaines, Christopher Costello

**Affiliations:** 1 Bren School of Environmental Science and Management, University of California, Santa Barbara, Santa Barbara, CA USA; 2 National Center for Ecological Analysis and Synthesis, University of California, Santa Barbara, Santa Barbara, CA USA; 3 Department of Geography, Florida State University, Tallahassee, FL USA; 4 Center for Coastal Marine Sciences, California Polytechnic State University, San Luis Obispo, CA USA

**Keywords:** Ecology, Ecology

## Abstract

Food security remains a principal challenge in the developing tropics where communities rely heavily on marine-based protein. While some improvements in fisheries management have been made in these regions, a large fraction of coastal fisheries remain unmanaged, mismanaged, or use only crude input controls. These quasi-open-access conditions often lead to severe overfishing, depleted stocks, and compromised food security. A possible fishery management approach in these institution-poor settings is to implement fully protected marine protected areas (MPAs). Although the primary push for MPAs has been to solve the conservation problems that arise from mismanagement, MPAs can also benefit fisheries beyond their borders. The literature has not completely characterized how to design MPAs under diverse ecological and economic conditions when food security is the objective. We integrated four key biological and economic variables (i.e., fish population growth rate, fish mobility, fish price, and fishing cost) as well as an important aspect of reserve design (MPA size) into a general model and determined their combined influence on food security when MPAs are implemented in an open-access setting. We explicitly modeled open-access conditions that account for the behavioral response of fishers to the MPA; this approach is distinct from much of the literature that focuses on assumptions of “scorched earth” (i.e., severe over-fishing), optimized management, or an arbitrarily defined fishing mortality outside the MPA’s boundaries. We found that the MPA size that optimizes catch depends strongly on economic variables. Large MPAs optimize catch for species heavily harvested for their high value and/or low harvesting cost, while small MPAs or no closure are best for species lightly harvested for their low value and high harvesting cost. Contrary to previous theoretical expectations, both high and low mobility species are expected to experience conservation benefits from protection, although, as shown previously, greater conservation benefits are expected for low mobility species. Food security benefits from MPAs can be obtained from species of any mobility. Results deliver both qualitative insights and quantitative guidance for designing MPAs for food security in open-access fisheries.

## Introduction

While large-scale industrial fisheries are increasingly well-managed, small-scale coastal fisheries in the developing tropics continue to be significantly overfished^1^. This has led to steady declines in biomass and has created grave food security concerns across the tropics, raising globally-relevant questions about how to reverse these trends. While one possible approach is simply to better manage these fisheries using traditional approaches, causal factors such as weak management institutions, poor rule of law, and unstable governments are unlikely to be quickly remedied in many of these locations. An alternative approach that is increasingly pursued is to implement fully protected MPAs, which prohibit extractive activities within a designated area. The idea is that, if well-designed, these MPAs may improve food security outcomes, even if mismanagement of fisheries outside the MPA continues. Indeed, MPAs are increasingly being used as a fisheries management and conservation tool to enhance fish biomass, improve fish catch for adjacent fisheries, protect fish populations from decline, and restore and preserve natural ecosystems both in developed and developing nations^2–16^.

Designing MPAs that will improve fishery, conservation, and food security outcomes requires understanding the effect of MPA protection on diverse target species, and how the magnitude of the effect may be moderated by MPA size. Previous theoretical models predict that: (1) low mobility species gain more conservation benefits from protection than high mobility species^17,18^ and (2) larger MPAs result in greater conservation benefits^17,19–21^, because vulnerability to fishing depends on the likelihood that individuals move outside of MPA borders. Additionally, (3) fisheries benefits are highest for species of intermediate mobility^18–20,22,23^ because high mobility species are not sufficiently protected by MPAs to improve future yields, while low mobility species do not have sufficient levels of spill-over to fished areas to improve future yields^18,19,22,24^. However, in some settings, empirical studies have not found a strong relationship between species mobility or MPA size and conservation or fisheries outcomes^9,25^.

One challenge with matching theory and empirical results is that previous modeling efforts on MPAs have focused on only one or two key variables at a time, often limiting predictive power. A failure to accurately forecast the response of target species based solely on a single characteristic, like species’ mobility, suggests the need for more complete, multivariate models^19^. In particular, MPA models rarely incorporate economics nor account for the management outside of the MPA (but see Hannesson^17^ and White *et al*.^26^) and often assume that fishing effort is unresponsive to economic incentives^17,21,27–31^.

In assessing the likely effects of an MPA on fishery or conservation outcomes, a crucial factor is how the fishery is managed outside the MPA. Most studies adopt one of three assumptions: first, many ecology-based papers focus on the “scorched earth” assumption that all fish outside the MPA will be harvested^16,32,33^. This provides a useful benchmark of the most extreme conservation challenge, although it exaggerates the challenge under a reasonable model of fishing costs for nearly all situations. Second, some authors have focused on optimized management outside the MPA^34,35^. This assumption is relevant when designing MPAs as part of an optimized fishery, but will be overly optimistic in the presence of poor fishery management. The third common approach assumes a fixed fishing mortality rate that persists, with or without an MPA^29,30,36,37^. This approach is perhaps a more realistic depiction for the developing tropics, but it only crudely accounts for the likely behavioral response of fishers following the implementation of MPAs^38^. An additional (fourth) approach that is reflective of many fisheries in developing countries, but rarely used in the MPA modeling literature, is to assume that fisheries are poorly-managed or unmanaged, so fishers behave as if they are under open access. In that setting, fishing effort responds in real time to the conditions present in the fishery, which are affected by the MPA. As conditions improve, fishers earn higher profit, which leads them to increase fishing effort in the fishable area. As conditions deteriorate, fishers lose profit, which leads them to decrease fishing effort.

Here, we developed a bioeconomic model of a fishery under open access, which is meant to represent a broad class of fisheries in the developing tropics. In that setting, we derived the MPA size that maximizes food security (catch), recognizing that individual fishers respond in real time to economic conditions in the fishery^17,39–41^. We then evaluated how the optimal MPA size depends on economic (fish price and fishing cost) and biological (species mobility and population growth rate) parameters. Because we focused explicitly on MPA size for food security in an open-access fishery, we were able to derive sharp analytical results. We showed that the optimized design of an MPA depends on the interplay between ecological and economic variables. For example, while a small MPA is generally preferred for fast growing, low-mobility species to allow sufficient movement of biomass to fishing areas, large MPAs optimize catch for such species when the species is of high economic value. These results both extend existing insights about the conditions under which MPAs can benefit fisheries, and provide guidance for estimating the optimal size of MPAs to achieve the objective of food security.

## Methods

**Figure 1:**
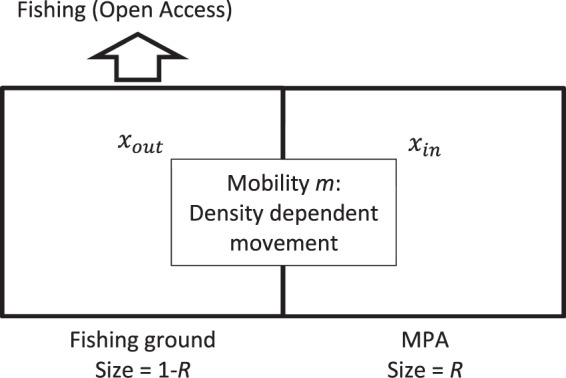
Schematic diagram of the model that investigates the role of biology, economics, and MPA size on MPA performance. The parameter *x* denotes fish population and *R* denotes MPA size. This model formulation is modified from ref.^30^.

We modeled the fishery as a two-patch system where one patch is fully protected and the other patch is open to fishing (Fig. 1). The two patches are biologically connected through density-dependent movement of harvestable fish population. Fishing dynamics are open-access, meaning that fishers adjust effort in response to profits, which are determined by fishing costs, fish price, and the biomass of the fish stock.

Mathematically, we modeled the fishery as coupled differential equations:

1 $$\frac{d{x}_{out}}{dt}=r{x}_{out}(1-\frac{{x}_{out}}{1-R})-qE{x}_{out}+m[(1-R){x}_{in}-R{x}_{out}]$$

2 $$\frac{d{x}_{in}}{dt}=r{x}_{in}(1-\frac{{x}_{in}}{R})-m[(1-R){x}_{in}-R{x}_{out}]$$

3 $$\frac{dE}{dt}=(pq{x}_{out}-c)E$$

where *x*_*out*_ is the fish population outside the MPA, *x*_*in*_ is the fish population inside the MPA, *r* is the growth rate of the fish population, *q* is the catchability of the fish, *E* is the fishing effort, *p* is the fish price per unit of catch, *c* is the fishing cost per unit of fishing effort, and *R* is the fraction of the species’ range in the protected area, i.e., the size of the MPA^19,42^ (see Table 1 for the ranges of parameter values used in our model). The last term in Eqs (1) and (2) contain the migration term. If the density inside the reserve is larger than the density outside, the term in square brackets is positive, so fish migrate outside. This term represents the “full movement biomass transfer” or the total amount of fish biomass that moves under instant equalization of biomass density. The parameter *m* is our movement parameter that dictates the speed of migration, defined as the fraction of the full movement biomass transfer (see Supplementary Information). *m* = 0 means that the species being modeled is sessile while *m* = 1 indicates a highly mobile species for which population density quickly equilibrates between the fished and protected areas. The main policy lever for a manager is *R*. Most of the analyses below focus on identifying the optimal value of *R*. We set the carrying capacity to 1 so that the fish populations *x*_*out*_ and *x*_*in*_ are relative to the carrying capacity and in the range from 0 to1. $\frac{d{x}_{out}}{dt}$, $\frac{d{x}_{in}}{dt}$, and $\frac{dE}{dt}$ are the rates of change of the fish populations outside and inside the MPA and of fishing effort, respectively. Equation (3) is the canonical equation describing how fishers allocate their fishing effort in response to fishery profitability in an open-access fishery. In that equation, fishers are assumed to increase or decrease effort in response to contemporaneous profit earned outside the MPA^40^. Fishing effort increases $(\frac{dE}{dt} > 0)$ whenever the total fishery profit is positive and decreases $(\frac{dE}{dt} < 0)\,\,$under negative profits. Note that the rate of increase or decrease in effort can be tuned by adding a scaling factor but that in steady-state this rate of effort change will become irrelevant.

**Table 1:** Parameters used in the MPA model.

Parameters	Values	Units	Description
*x*_out_	Dynamic. Can take any value from 0–1.	Unitless. We assumed that biomass is relative to the total fish population carrying capacity so that with no fishing, total biomass is equal to 1	Biomass outside Marine Protected Area (MPA)
*x*_in_	Dynamic. Can take any value from 0–1.	Unitless. Same explanation as *x*_out_	Biomass inside MPA
*R*	[0,1]	Relative to species stock range	MPA size
*r*	[0.001,1]	per unit time, e.g. Year^−1^	Species growth rate
*m*	[0.01,1]	per unit time, e.g. Year^−1^	Fish mobility
*E*	Dynamic	unit of effort (e.g. number of fishers, fishing days, number of hooks)	Fishing effort
*q*	—	fishing mortality per unit of effort	Catchability coefficient
*c*	—	e.g. USD/unit of fishing mortality	Variable fishing cost per unit of fishing mortality
*p*	—	e.g. USD/MT	Ex-vessel fish price
c/pq	[0,0.5]	e.g. MT	The ratio of fishing cost to the product of fish price and catchability coefficient is taken as a single parameter

We derived the steady-state biomass and catch of a fish population under different growth rates and movement rates for different MPA sizes. In steady state, the rate of change in biomass and fishing effort in Eqs (1–3) will be 0:

4 $$r{x}_{out}^{\ast }(1-\frac{{x}_{out}^{\ast }}{1-R})-q{E}^{\ast }{x}_{out}^{\ast }+m[(1-R){x}_{in}^{\ast }-R{x}_{out}^{\ast }]=0$$

5 $$r{x}_{in}^{\ast }(1-\frac{{x}_{in}^{\ast }}{R})-m[(1-R){x}_{in}^{\ast }-R{x}_{out}^{\ast }]=0$$

6 $${E}^{\ast }(pq{x}_{out}^{\ast }-c)=0$$

where ${x}_{in}^{\ast }$ and ${x}_{out}^{\ast }$ are the steady-state fish biomass inside and outside the MPA after all ecological and economic adjustments have occurred. From Eq. (6), for a fishery to be in steady state, either ${E}^{\ast }=0$ or $pq{x}_{out}^{\ast }-c=0$. If ${E}^{\ast }=0$, then the fishery is not profitable to fish, so the steady-state biomass outside the MPA is ${x}_{out}^{\ast }=1-R$. But the more typical case is when ${E}^{\ast } > 0$, which implies the fish stock outside the reserve is:

7 $${x}_{out}^{\ast }=\frac{c}{pq}$$

We derived the fish stock inside the reserve by solving Eq. (5) for ${x}_{in}^{\ast }$:

8 $${x}_{in}^{\ast }=\pm \frac{R\sqrt{4mr{x}_{out}^{\ast }+{m}^{2}+{r}^{2}+{m}^{2}{R}^{2}-2mr-2{m}^{2}R+2mrR}-(mR-rR-m{R}^{2})}{2r}$$

Only the real and positive solution will be accepted.

Finally, it is possible to derive the fishing effort in steady state by rearranging Eq. (4) as follows:

9 $${E}^{\ast }=\,\frac{r{x}_{out}^{\ast }(1-\frac{{x}_{out}^{\ast }}{1-R})+m[(1-R){x}_{in}^{\ast }-R{x}_{out}^{\ast }]}{q{x}_{out}^{\ast }}$$

The total steady-state biomass is:

10 $${B}^{\ast }={x}_{out}^{\ast }+{x}_{in}^{\ast }$$

The steady-state catch, which we used as a direct measure of food security in the fishery, is:

11 $${H}^{\ast }=q{E}^{\ast }{x}_{out}^{\ast }\,$$

When fish stock dynamics are represented by a logistic growth function, fishing at maximum sustainable yield (MSY) results in fish biomass that is half of the unfished biomass. In that simple benchmark case, with no MPA (*R* = 0), ${x}_{out}^{\ast }={B}^{\ast }=\frac{c}{pq}=0.5$ implies that the population is being fished at MSY (see Eq. (7)). In other words, the only way an open-access fishery without an MPA can maximize food provision is if $\frac{c}{pq}=\mathrm{0.5.}\,\,$If $\frac{c}{pq}$ is lower than that (e.g., if fish price is higher or fishing costs are lower), then biomass will be too low, and fish catch will be compromised. If $\frac{c}{pq}$ is higher (for example, if fishing costs are higher or fish prices are lower), then biomass will be too high, and fish catch will be compromised. Either way, the fishery will yield lower than maximal potential fish catch.

We compared food security (fish catch) and conservation (fish biomass) performance without an MPA and with an MPA of different sizes at steady-state conditions.

### Optimal MPA size that maximizes catch

The optimal MPA size that maximizes catch can be derived by taking the partial derivative of the steady-state catch (Eq. (11)) with respect to *R.* However, the partial derivative is only smooth (meaning an analytic solution exists) with a harvesting cost of zero, i.e. *c* = 0, ${x}_{out}^{\ast }=0$. When harvesting cost is zero, open access results in the “scorched earth” scenario outside the MPA, providing a convenient benchmark with an analytical solution. For zero harvesting cost, the reserve size that maximizes catch under open access can be found as follows:

12 $$\frac{\partial {H}^{\ast }}{\partial R}=0$$

Solving Eq. (12) for *R* and renaming it *R*_opt_ to represent the optimal reserve size that maximizes catch (see Supplementary Information, Eq. (S5)), we have:

13 $${R}_{opt}{|}_{c=0}=\frac{2m-r+\sqrt{{m}^{2}-mr+{r}^{2}}}{3m}$$

For the case where *c* > 0, we derived the optimal reserve size that maximizes catch (*R*_opt_) by solving Eq. (12) numerically.

### Fish mobility and growth rate that produce maximum catch for different MPA sizes and economic condition

While fish mobility is not a policy choice per se, it is interesting to ask how the movement parameter affects food security for any given MPA size. The fish mobility that produces maximum catch (*m*_*opt*_) for different sizes of MPAs under open access can be derived by taking the partial derivative of the steady-state catch (Eq. (11)) with respect to the mobility term, and equating this to zero:

14 $$\frac{\partial {H}^{\ast }}{\partial m}=0$$

Solving for *m* and renaming it *m*_*opt*_ (to represent the fish mobility that produces the highest catch from protection) (see Supplementary Information, Eq. (S8)), we obtain:

15 $${m}_{opt}=\frac{r}{2(1-R-2\frac{c}{pq})}$$

To derive the relationship between growth rates and harvest for different MPA sizes, economic conditions, and fish mobility, we noted that the steady-state harvest term in Eq. (11) is a linear function of a species’ population growth rate. Therefore, holding MPA size, economic condition, and fish mobility constant, the higher the species growth rate, the higher the catch rate.

## Results

### MPA size that maximizes catch

**Figure 2:**
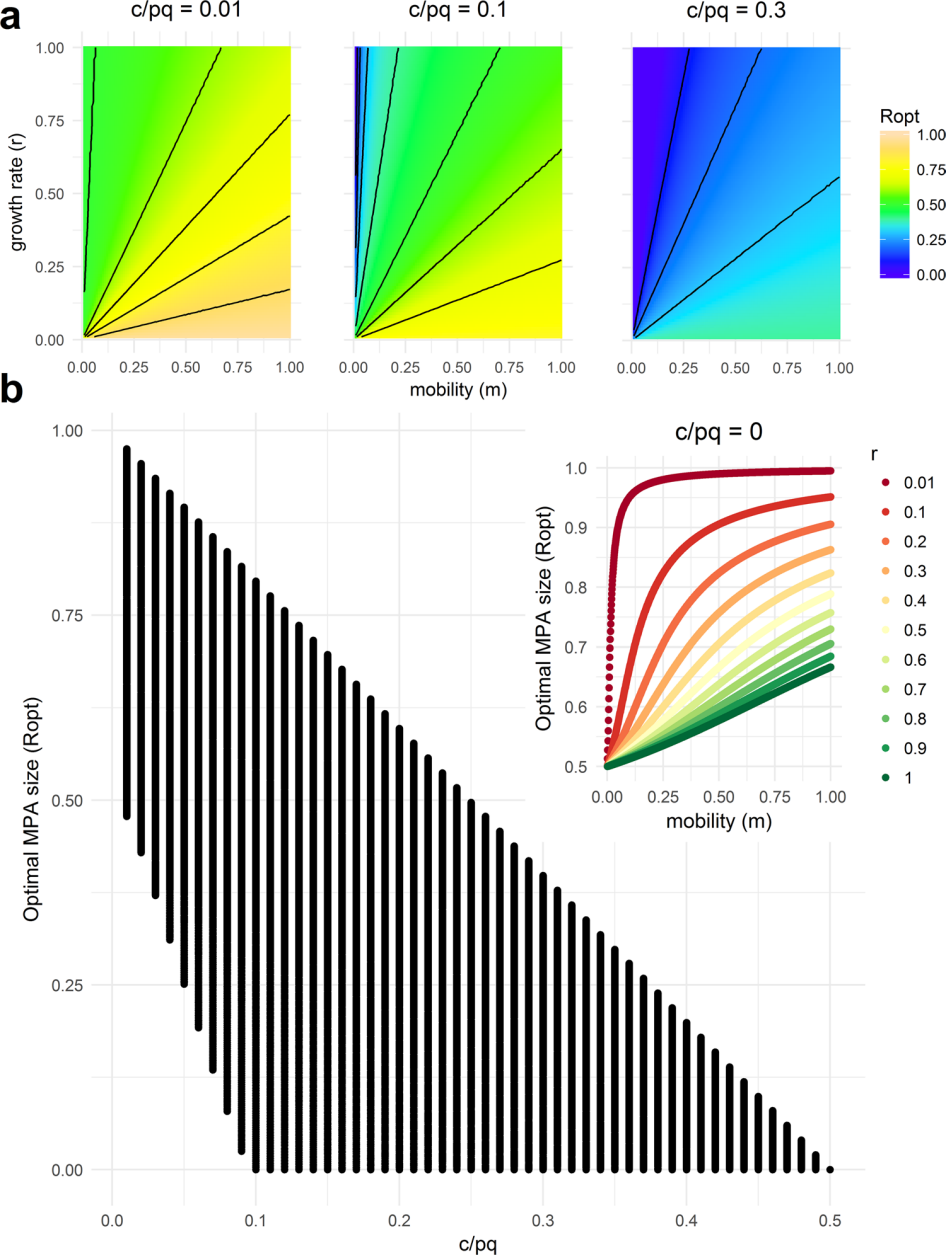
Optimal MPA size that maximizes catch (*R*_opt_) for variable growth rate ($r\in [0.01,\,1]$), mobility ($m\in [0.01,\,1]$), and cost-price catchability ratio $(c/pq\in [0,\,0.5])$. (**a**) The lines are contour lines of *R*_opt_ in increments of 0.1. (**b**) The same as (**a**) but projected into *R*_opt_ vs. *c*/*pq*. Inset represents *R*_opt_ for variable growth rate (*r*) and mobility (*m*) under the extreme yet commonly assumed case of zero cost of harvesting (*c*/*pq* = 0).

The MPA size that maximizes catch (*R*_opt_) strongly depends on economic parameters (i.e., the cost-price ratio, $\frac{c}{pq}$) (Fig. 2), because these parameters indicate the incentives to fish more or less aggressively outside the MPA. Larger MPAs are optimal for species heavily targeted for their low harvesting cost or high market price (lower values of $\frac{c}{pq}$), while small MPAs are optimal for higher values of $\frac{c}{pq}$, until $\frac{c}{pq}\ge 0.5$, at which point establishing an MPA is not optimal regardless of the biological characteristics of the species (Fig. 2). This latter result occurs when harvesting costs are sufficiently high (or fish prices are sufficiently low) and fishers naturally reduce fishing pressure below what would be required to maximize catch as fishing at MSY is not economically viable. Implementing an MPA in that setting would only further reduce catch. While this scenario is of theoretical interest, we regard it as unlikely in the applied settings we aim to inform, since there would be no conservation challenge to solve even in the absence of the MPA.

Consistent with the previous literature, we found that more mobile species require a larger MPA size in order to maximize catch (Fig. 2a). We also found that the higher the species’ population growth rate, the smaller the optimal MPA size. Therefore, for a specific $\frac{c}{pq}$ value (where $\frac{c}{pq} < 0.5)$, larger MPAs are often required to optimize catch for high mobility, slow-growing species, whereas smaller MPAs are often required for low mobility, fast-growing species (Fig. 2a).

For the limiting case of $\frac{c}{pq}=0$, i.e., there is no cost of harvesting and everything outside the reserve is harvested (e.g., ref.^32^), the optimal MPA size is bounded between 50% and 100% of the species’ stock range (Fig. 2b and inset). As mobility approaches zero, the optimal MPA size approaches 50% of the stock range (Fig. 2b inset).

### Effect of biological and economic parameters on the fisheries and conservation benefit of MPA

**Figure 3:**
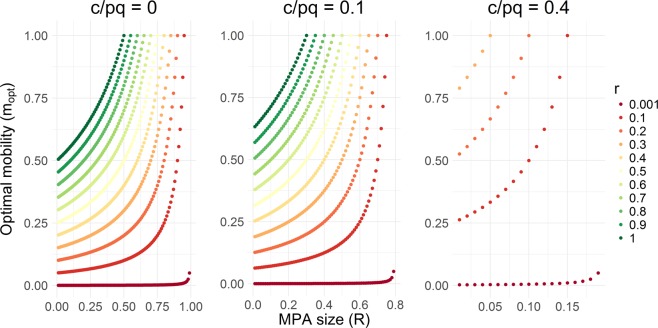
Optimal species mobility that maximizes catch for different MPA sizes (*R*), growth rates (*r*), and cost-price catchability ratios (*c*/*pq*).

For any conditions where fishing is economically rational, the higher the species’ population growth rate, the higher the catch rate. We also showed that the species mobility that is favored to produce maximum catch is influenced by the species growth rate (*r*), economic variables $(\frac{c}{pq})$, and MPA size (*R*). In particular, the species mobility that is favored to produce maximum catch increases linearly with increasing species’ population growth rate (Eq. (15)) and non-linearly with increasing MPA size and decreasing exploitation level (as determined by the economic variables $\frac{c}{pq}$) (Fig. 3). Contrary to previous theoretical results, low- and high-mobility species can be optimal fisheries targets (i.e., for improving food security). In general, a combination of low values of *r*, *R*, and $\frac{c}{pq}$ favors low mobility species as fisheries targets, while a combination of high values of *r*, *R*, and $\frac{c}{pq}$ favors high mobility species as fisheries targets.

**Figure 4:**
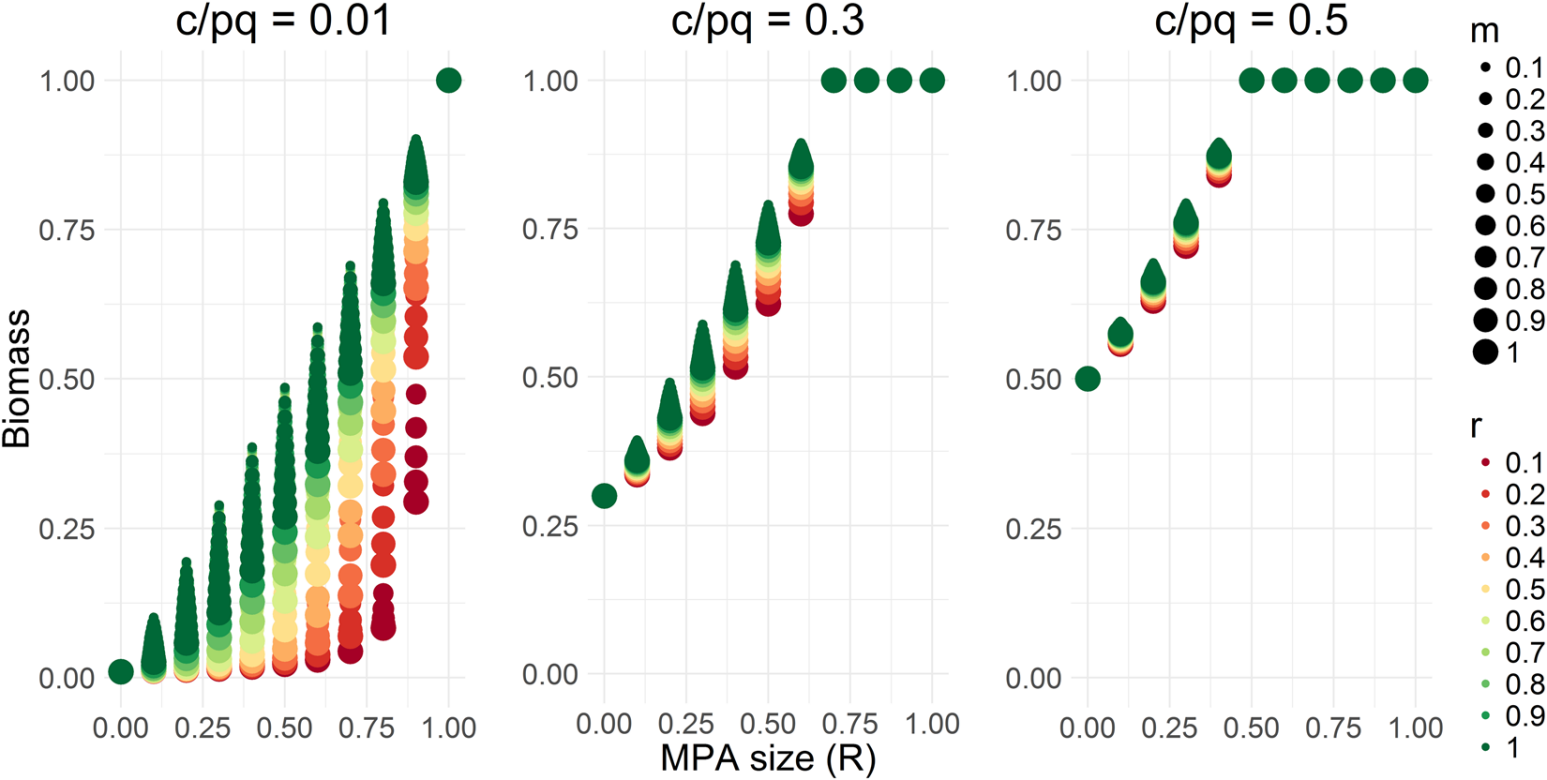
Steady-state biomass for different levels of MPA size (*R*), growth rate (*r*), mobility (*m*), and cost-price catchability ratio (*c*/*pq*).

Examining conservation (i.e., steady-state biomass) effects, we found that the difference in biomass between a fished population with and without an MPA depends on the economic and biological parameters and MPA size. Controlling for the interacting parameters, i.e. assuming constant values, we found that low-mobility species are expected to gain more conservation benefit from protection than high-mobility species (Fig. 4). Similarly, fast-growing species are expected to gain more conservation benefit from protection than slow-growing species.

For $\frac{c}{pq}$ close to 0 (i.e., for species heavily targeted for their high price or low harvesting cost), the conservation benefit of protection is much greater for low-mobility species than high-mobility species and for fast-growing species than slow-growing species (Fig. 4). As $\frac{c}{pq}$ increases, the difference in the conservation benefit of protection for different biological characteristics narrows. This is because species become less economically viable to fish, resulting in a low harvest rate, regardless of their biological characteristics (Fig. 4). This highlights the interesting and important interplay between economic and ecological parameters in determining the consequences of MPAs in open-access fisheries.

## Discussion

Fisheries in the developing tropics are increasingly overfished, which raises serious concerns about food security for vulnerable populations. We developed a bioeconomic model to inform the design of MPAs in governance-poor, open-access fisheries with the explicit goal of maximizing food provision from the adjacent fisheries. Our model also explicitly accounted for fishers’ behavior outside the MPA, where we assumed fishing effort responds in real time to economic profitability. Analyzing this model allowed us to gain insights into the impact of the fisheries’ economics, the biology of the target species, and MPA size on MPA performance, for both conservation and fisheries objectives.

Past models of MPA performance have rarely incorporated rational economic behavior^21,24,27,28,31^; this can lead to biased estimates of the food-provision consequences of MPA establishment. This gap in the literature is surprising given an appreciation for the role of fishing mortality in determining MPA performance, the potential tradeoff between conservation and fisheries goals^43,44^, and the effects of economics in influencing fishing pressure^17,45^.

**Figure 5:**
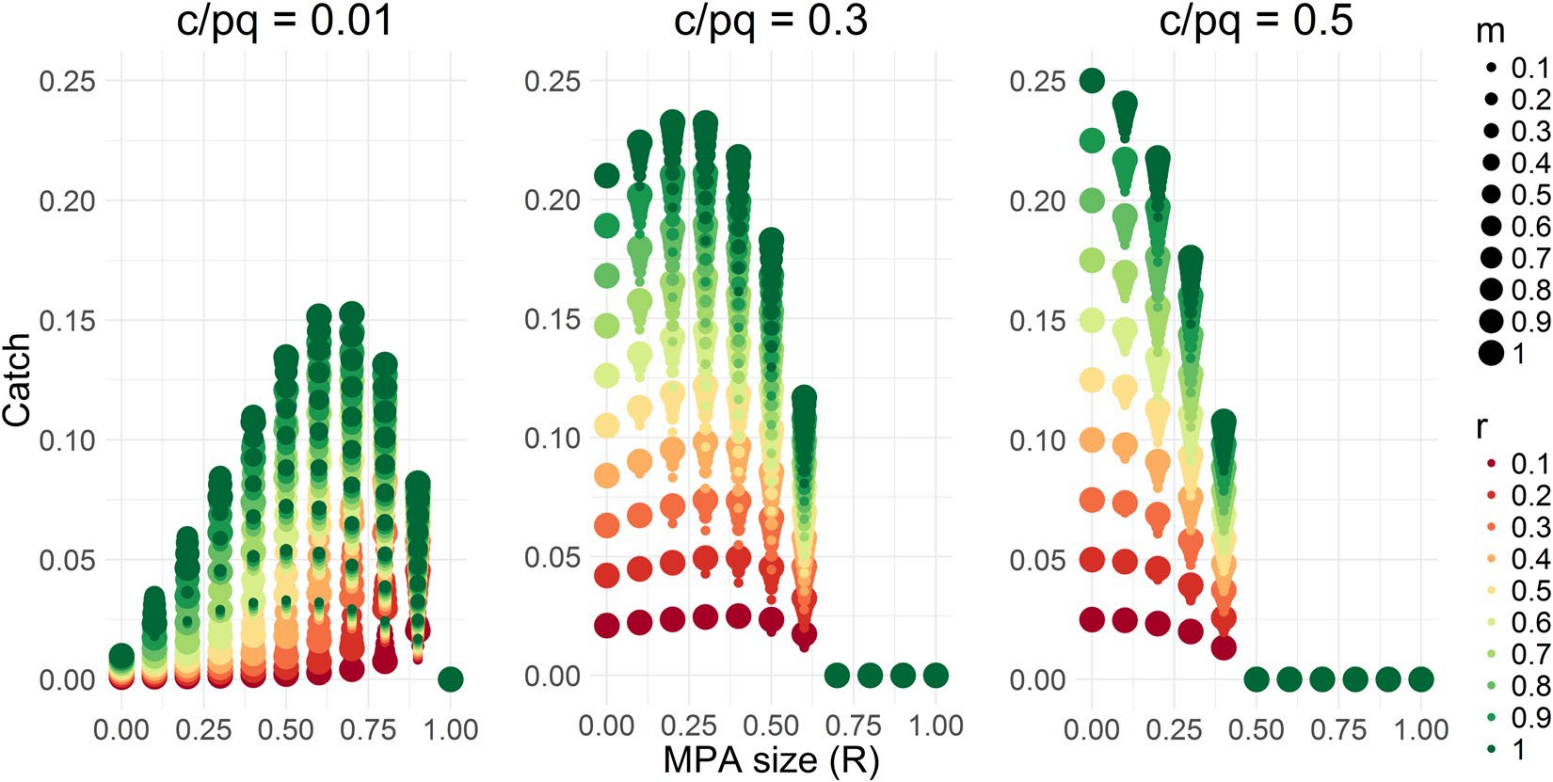
Steady-state fish catch for different levels of fish mobility (*m*), population growth rate (*r*), cost-price catchability ratio (*c/pq*), and MPA size (*R*).

We found that the economic parameters governing fishers’ behavior can strongly influence optimal MPA size for different biological characteristics of the target species. Generally, when economic conditions lead to severe overfishing in the absence of MPAs, large MPAs (>50%) can significantly improve both conservation and food provision^21,46^ (Fig. 2b). On the other hand, for species that are lightly to moderately fished (for example, due to low prices or high harvesting cost), small- to moderately-sized MPAs are needed to optimize food provision (Fig. 2b, Fig. 5).

We show that, contrary to some previous predictions, fishery benefits from establishing an MPA are not always highest for species of intermediate mobility^18–20,22,23^. Maximum fishery benefits are also possible for fisheries targeting low-mobility or very vagile species, a result supported by previous empirical studies^9,25,47^. The economics of the fisheries, species’ population growth rate, and MPA size play key roles in determining the species mobility level that is expected to gain optimal fisheries benefits from protection. For example, optimal fisheries benefits can be obtained from highly mobile species that are heavily targeted for their high economic value, such as the North Sea cod, by establishing large MPAs (>50% of the total fished area)^21^. The high mobility of the species enables fish stocks to spill-over to fishing areas, while the large size of the MPA controls the amount of area available for fishing. Optimal fisheries benefits can be obtained for low-mobility species such as the California spiny lobster^48^ and the European lobster^49^ by establishing small- to moderately-sized MPAs. The small- to moderately-sized MPAs ensure sufficient movement of low-mobility species to fishing areas where they can benefit fish catch.

We found general support for the theoretical prediction that low-mobility species should experience greater conservation benefit from protection^17,18^. However, our results contradict the theoretical prediction that highly mobile species cannot benefit from MPAs, especially when the MPA is large and the species is fast-growing^17,19,21^. Empirical work has also found that high-mobility species can benefit^50^, although this may in part be a result of site fidelity behavior that limits their mobility while in the MPA, which is not included in our model.

Controlling for other parameters, the conservation benefit from protection is greatest for larger MPAs and for fast-growing species, as expected. The differences in conservation benefits for different biological characteristics are more pronounced for heavily targeted species (for example, for those with low harvesting costs or high prices, see $\frac{c}{pq}=0.01$, Fig. 4). However, these differences narrow with increasing $\frac{c}{pq}$ values. Over half of the species included in the meta-analyses conducted by Micheli *et al*.^9^ and Claudet *et al*.^25^ have high $\frac{c}{pq}$ or are lightly to moderately targeted species, potentially explaining the similar conservation responses to protection of low-, moderate-, and high-mobility species. The heterogeneity in the size of the MPAs, species growth rates, management regime in fishing areas, and intensity of fishing pressure for the studies included in these meta-analyses make it difficult to derive general predictions, highlighting the utility of a multifactor bioeconomic model, such as the one developed here.

Our model relied on a number of general but defensible assumptions and simplifications that influenced our results. We assumed fish migration is density-dependent^11,30,51,52^, which may neglect species adaptive behavior to protection, such as high site fidelity of species inside MPAs^53^. Furthermore, we assumed that the protected zone and the fishing zone are adjacent and continuous and that species move freely in these two areas. Our model also assumed that the population is closed, which will not hold in all contexts. The cost function for catching fish is also uniform outside the MPA, ignoring the potential benefit of fishing-the-line^54^ or benefits from larval export from the MPA. A straightforward extension of this work could include larval exchange dynamics among metapopulations to address questions such as fisheries and conservation effects of MPA networks^33,55,56^. Fish population build-up in protected ‘source’ zones can contribute larvae to fished ‘sink’ sites, enhancing the productivity of those sites.

A particularly important assumption here is that the fishery is poorly managed, so open-access dynamics prevail with no other management aside from an MPA. This condition is common in the developing world^5,57,58^ and captures the class of low-governance fisheries that may be most concerned with food security, but where fishing effort responds in real time to economic returns. In the Philippines, for example, where fisheries are generally open access, MPAs may be expected to increase conservation and possibly fish catch, but may not show any improvements in fisheries profit. Indeed, a recent study conducted in the Philippines showed that MPAs increased fish biomass and density within MPA borders but did not improve fishery profit^59^. In our model, since the fishery outside the MPA remains open access, fishing effort ramps up so that in steady state, no profits persist; this result holds regardless of the MPA size. While an MPA cannot increase steady state profit in an open-access fishery, our main result is that MPAs can indeed be optimized to increase food provision, even for purely open-access fisheries. MPAs may be attractive to planners in this context as they are straightforward to design and may be easier to implement and enforce compared to fisheries regulations such as catch or size limits. Other management options outside the MPA, such as Territorial Use Rights for Fisheries, may provide options to enhance both food security and livelihoods^60,61^.

Our paper currently focuses on modeling MPA effects on single species. Evaluating MPA effects in a multispecies context is an interesting and important extension. This requires tracking individual species’ population biomass inside and outside MPA, accounting for interactions among different species^16^, as well as modeling how fishers redistribute their fishing effort among different target species. Future modeling effort should look into how MPAs affect multiple interacting species that have different biological and economic characteristics. It is expected that no single MPA size will be simultaneously optimal for a well-mixed, multi-species population as different species respond differently from MPAs. Accounting for habitat heterogeneity and species distribution in a multi-species context allows for the design of MPAs that can simultaneously improve harvest and conservation for a variety of species. While the current results of our models cannot be directly interpreted in a multi-species context, the framework developed here could be extended to represent a multi-species context.

Millions of fishers harvesting in thousands of fisheries all over the world attempt to maintain a livelihood and sustenance from open-access fisheries. While entry and exit dynamics depend on economic returns, we asked whether MPAs can be designed to increase food security for this important class of fisheries. By developing and analyzing a theoretical bioeconomic model, we showed how the interaction of the economic conditions that drive exploitation rates of the target species, species biology, and size of an MPA determine the food security and conservation effects of an MPA. Neglecting these interactions is likely to result in unrealistic expectations of fishery and conservation benefits from establishing MPAs.

## Supplementary information

Supplementary Information

## Data Availability

No datasets were generated or analysed during the current study.

## References

[1] Costello C (2012). Status and solutions for the world’s unassessed fisheries. Science.

[2] Dugan JE, Davis GE (1993). Applications of marine refugia to coastal fisheries management. Can. J. Fish. Aquat. Sci..

[3] Allison GW, Lubchenco J, Carr MH (1998). Marine reserves are necessary but not sufficient for marine conservation. Ecol. Appl..

[4] Roberts CM, Bohnsack JA, Gell F, Hawkins JP, Goodridge R (2001). Effects of marine reserves on adjacent fisheries. Science.

[5] Alcala AC, Russ GR (2006). No-take marine reserves and reef fisheries management in the Philippines: A new people power revolution. AMBIO J. Hum. Environ..

[6] Worm B (2006). Impacts of biodiversity loss on ocean ecosystem services. Science.

[7] Gaines SD, Lester SE, Grorud-Colvert K, Costello C, Pollnac R (2010). Evolving science of marine reserves: New developments and emerging research frontiers. Proc. Natl. Acad. Sci..

[8] Halpern BS (2003). The impact of marine reserves: Do reserves work and does reserve size matter?. Ecol. Appl..

[9] Micheli F, Halpern BS, Botsford LW, Warner RR (2004). Trajectories and correlates of community change in no-take marine reserves. Ecol. Appl..

[10] Russ GR, Alcala AC, Maypa AP, Calumpong HP, White AT (2004). Marine reserve benefits local fisheries. Ecol. Appl..

[11] Halpern BS, Lester SE, Kellner JB (2009). Spillover from marine reserves and the replenishment of fished stocks. Environ. Conserv..

[12] Lester SE (2009). Biological effects within no-take marine reserves: A global synthesis. Mar. Ecol. Prog. Ser..

[13] Caselle JE, Rassweiler A, Hamilton SL, Warner RR (2015). Recovery trajectories of kelp forest animals are rapid yet spatially variable across a network of temperate marine protected areas. Sci. Rep..

[14] Hooker SK, Gerber LR (2004). Marine reserves as a tool for ecosystem-based management: The potential importance of megafauna. Bio Science.

[15] Halpern BS, Lester SE, McLeod KL (2010). Placing marine protected areas onto the ecosystem-based management seascape. Proc. Natl. Acad. Sci..

[16] Hastings, A., Gaines, S. D. & Costello, C. Marine reserves solve an important bycatch problem in fisheries. *Proc*. *Natl*. *Acad*. *Sci*. 201705169 (2017). 10.1073/pnas.1705169114 PMC5576807 28794280

[17] Hannesson R (1998). Marine reserves: What would they accomplish?. Mar. Resour. Econ..

[18] Botsford LW, Micheli F, Hastings A (2003). Principles for the design of marine reserves. Ecol. Appl..

[19] Palumbi SR (2004). Marine reserves and ocean neighborhoods: The spatial scale of marine populations and their management. Annu. Rev. Environ. Resour..

[20] Hilborn R (2004). When can marine reserves improve fisheries management?. Ocean Coast. Manag..

[21] Le Quesne WJF, Codling EA (2009). Managing mobile species with MPAs: The effects of mobility, larval dispersal, and fishing mortality on closure size. ICES J. Mar. Sci..

[22] Roberts, C. & Hawkins, J. *Fully-protected marine reserves: A guide*. (WWF Endangered Seas Campaign, 2000).

[23] Gell FR, Roberts CM (2003). Benefits beyond boundaries: the fishery effects of marine reserves. Trends Ecol. Evol..

[24] Polacheck T (1990). Year around closed areas as a management tool. Nat. Resour. Model..

[25] Claudet J (2010). Marine reserves: Fish life history and ecological traits matter. Ecol. Appl..

[26] White C, Kendall BE, Gaines S, Siegel DA, Costello C (2008). Marine reserve effects on fishery profit. Ecol. Lett..

[27] Guénette S, Pitcher TJ (1999). An age-structured model showing the benefits of marine reserves in controlling overexploitation. Fish. Res..

[28] Apostolaki P, Milner-Gulland EJ, McAllister MK, Kirkwood GP (2002). Modelling the effects of establishing a marine reserve for mobile fish species. Can. J. Fish. Aquat. Sci..

[29] Rodwell LD, Barbier EB, Roberts CM, McClanahan TR (2002). A model of tropical marine reserve-fishery linkages. Nat. Resour. Model..

[30] Takashina N, Mougi A, Iwasa Y (2012). Paradox of marine protected areas: suppression of fishing may cause species loss. Popul. Ecol..

[31] Barnes B, Sidhu H (2013). The impact of marine closed areas on fishing yield under a variety of management strategies and stock depletion levels. Ecol. Model..

[32] Hastings A, Botsford LW (1999). Equivalence in yield from marine reserves and traditional fisheries management. Science.

[33] Cabral RB (2016). Siting marine protected areas based on habitat quality and extent provides the greatest benefit to spatially structured metapopulations. Ecosphere.

[34] Sanchirico JN, Malvadkar U, Hastings A, Wilen JE (2006). When are no-take zones an economically optimal fishery management strategy?. Ecol. Appl..

[35] Costello C, Polasky S (2008). Optimal harvesting of stochastic spatial resources. J. Environ. Econ. Manag..

[36] Carr MH, Reed DC (1993). Conceptual issues relevant to marine harvest refuges: Examples from temperate reef fishes. Can. J. Fish. Aquat. Sci..

[37] Man A, Law R, Polunin NVC (1995). Role of marine reserves in recruitment to reef fisheries: A metapopulation model. Biol. Conserv..

[38] Cabral RB, Gaines SD, Johnson BA, Bell TW, White C (2017). Drivers of redistribution of fishing and non-fishing effort after the implementation of a marine protected area network. Ecol. Appl..

[39] Pezzey JCV, Roberts CM, Urdal BT (2000). A simple bioeconomic model of a marine reserve. Ecol. Econ..

[40] Sanchirico JN, Wilen JE (2001). A bioeconomic model of marine reserve creation. J. Environ. Econ. Manag..

[41] Hilborn R, Micheli F, De Leo GA (2006). Integrating marine protected areas with catch regulation. Can. J. Fish. Aquat. Sci..

[42] Kramer DL, Chapman MR (1999). Implications of fish home range size and relocation for marine reserve function. Environ. Biol. Fishes.

[43] Cabral RB, Halpern BS, Costello C, Gaines SD (2017). Unexpected management choices when accounting for uncertainty in ecosystem service tradeoff analyses. Conserv. Lett..

[44] Rassweiler A, Costello C, Hilborn R, Siegel DA (2014). Integrating scientific guidance into marine spatial planning. Proc. R. Soc. Lond. B Biol. Sci..

[45] Costello C (2016). Global fishery prospects under contrasting management regimes. Proc. Natl. Acad. Sci..

[46] Hart DR (2006). When do marine reserves increase fishery yield?. Can. J. Fish. Aquat. Sci..

[47] Horta e Costa B, Erzini K, Caselle J, Folhas H, Gonçalves E (2013). ‘Reserve effect’ within a temperate marine protected area in the north-eastern Atlantic (Arrábida Marine Park, Portugal). Mar. Ecol. Prog. Ser..

[48] Kay MC, Lenihan HS, Kotchen MJ, Miller CJ (2012). Effects of marine reserves on California spiny lobster are robust and modified by fine-scale habitat features and distance from reserve borders. Mar. Ecol. Prog. Ser..

[49] Moland E (2013). Lobster and cod benefit from small-scale northern marine protected areas: Inference from an empirical before–after control-impact study. Proc R Soc B.

[50] Koldewey HJ, Curnick D, Harding S, Harrison LR, Gollock M (2010). Potential benefits to fisheries and biodiversity of the Chagos Archipelago/British Indian Ocean Territory as a no-take marine reserve. Mar. Pollut. Bull..

[51] Abesamis RA, Russ GR (2005). Density-dependent spillover from a marine reserve: Long-term evidence. Ecol. Appl..

[52] Kellner JB, Nisbet RM, Gaines SD (2008). Spillover from marine reserves related to mechanisms of population regulation. Theor. Ecol..

[53] Grüss A, Kaplan DM, Guénette S, Roberts CM, Botsford LW (2011). Consequences of adult and juvenile movement for marine protected areas. Biol. Conserv..

[54] Kellner JB, Tetreault I, Gaines SD, Nisbet RM (2007). Fishing the line near marine reserves in single and multispecies fisheries. Ecol. Appl..

[55] Hastings A, Botsford LW (2003). Comparing designs of marine reserves for fisheries and for biodiversity. Ecol. Appl..

[56] Gaines SD, White C, Carr MH, Palumbi SR (2010). Designing marine reserve networks for both conservation and fisheries management. Proc. Natl. Acad. Sci..

[57] Cabral RB (2014). The Philippine marine protected area (MPA). database. Philipp. Sci. Lett..

[58] Muallil RN, Mamauag SS, Cabral RB, Celeste-Dizon EO, Aliño PM (2014). Status, trends and challenges in the sustainability of small-scale fisheries in the Philippines: Insights from FISHDA (Fishing Industries’ Support in Handling Decisions Application) model. Mar. Policy.

[59] Ferrer, A. J. G., Francisco, H. A., Carmelita, B. M. M. & Hopanda, J. C. *How do marine protected areas affect the welfare of local fishing communities? A study from the Philippines*. (Economy and Environment Program for Southeast Asia, 2017).

[60] Wilen JE, Cancino J, Uchida H (2012). The economics of territorial use rights fisheries, or TURFs. Rev. Environ. Econ. Policy.

[61] Gelcich S, Donlan CJ (2015). Incentivizing biodiversity conservation in artisanal fishing communities through territorial user rights and business model innovation. Conserv. Biol..

