# Supplementary material for: Designing MPAs for food security in open-access fisheries
Source: Sci Rep. 2019 May 29;9:8033. doi: 10.1038/s41598-019-44406-w (PMC6541654; doi:10.1038/s41598-019-44406-w)
Supplement: Supplementary file 1 — Supplementary Information [file 41598_2019_44406_MOESM1_ESM.docx]

**Supplementary Information**

Designing MPAs for food security in open-access fisheries

Reniel B. Cabral^1^*, Benjamin S. Halpern^1,2^, Sarah E. Lester^3^, Crow White^4^, Steven D. Gaines^1^, Christopher Costello^1^

^1^ Bren School of Environmental Science and Management, University of California, Santa Barbara, Santa Barbara, CA, USA

^2^ National Center for Ecological Analysis and Synthesis, University of California, Santa Barbara, Santa Barbara, CA, USA

^3^ Department of Geography, Florida State University, FL, USA

^4^ Center for Coastal Marine Sciences, California Polytechnic State University, San Luis Obispo, CA, USA

* email: [rcabral@bren.ucsb.edu](mailto:rcabral@bren.ucsb.edu)

**Derivation of fish migration term**

The “full movement biomass transfer” or the total amount of fish biomass that moves from inside MPA to outside under instant equalization of biomass density ($\Delta x$) can be derived using the following relationship:

| $\frac{x_{in}-\Delta x}{R}=\frac{x_{in}+\Delta x}{1-R}$ | (S1) |
| --- | --- |

i.e., the fish density inside the MPA equals the density outside after fish transfer. $x_{in}$ is the total biomass inside the MPA, $x_{out}$ is the total biomass in fishing area, and *R* is the MPA size. Solving for $\Delta x$, we have:

| $\Delta x=\left( 1-R \right)x_{in}-Rx_{out}$ | (S2) |
| --- | --- |

We actually do not want the full $\Delta x$ to transfer from inside to outside MPA as species have different movement rates. Rather, for some species, a fraction of $\Delta x$ (which we denote as *m*) moves from inside to outside MPA. The parameter *m* is our movement parameter that dictates the speed of migration. *m*=0 represents the movement of sessile species while *m*=1 represents highly mobile species for which population density quickly equilibrates between the fished and protected areas. Our fish migration term is then:

| $m\left[ \left( 1-R \right)x_{in}-Rx_{out} \right]$ | (S3) |
| --- | --- |

**MPA size that optimizes catch**

The MPA size that optimizes catch is expressed in Equation (12):

| $\frac{\partial H^{*}}{\partial R}=0$ | (S4) |
| --- | --- |
| Using *H** in equation (11) and taking the partial derivative of *H** with respect to *R*, we have:  $\left( 3mR^{2}+\left( 2r-4m \right)R-r+m \right)\left( \sqrt{m^{2}R^{2}+2mrR-2m^{2}R+r^{2}-2mr+m^{2}}+mR+r-m \right)=0$ | (S5) |

Renaming *R* to *R*_opt_ to represent the optimal MPA size that maximizes catch, we derive equation (13).

**Fish mobility that produces maximum catch**

The fish mobility the produces maximum catch for different sizes of MPAs under open access is expressed in Equation (14):

| $\frac{\partial H^{*}}{\partial m}=0$ | (S6) |
| --- | --- |

Using *H** in equation (11), we have

| $\frac{\partial}{\partial m}\left( m\left( 1-R \right)x_{in}^{*} \right)-Rx_{out}^{*}=0$ | (S7) |
| --- | --- |

or

| $x_{out}^{*}+\frac{\left( R-1 \right)\left( \begin{aligned} \left( \left( 2R-2 \right)m+r \right)\sqrt{R^{2}m^{2}-2Rm^{2}+m^{2}+4rx_{out}^{*}m+{2Rrm-2rm+r}^{2}}\pm\\ \left( 2R^{2}-4R+2 \right)m^{2}\pm\left( 6x_{out}^{*}+3R-3 \right)rm\pm r^{2} \end{aligned} \right)}{2r\sqrt{R^{2}m^{2}-2Rm^{2}+m^{2}+4rx_{out}^{*}m+2Rrm-2rm+r^{2}}}=0.$ | (S8) |
| --- | --- |
